# Book Reviews

**Published:** 1905-03

**Authors:** 


Manual of Physiological and Clinical Chemistry. By Elias H. Bartley, M.D., Ph.G., Professor of Chemistry, Toxicology and Pediatrics in the Long Island College Hospital. Second edition, revised and enlarged. Duodecimo, 188 pages. Illustrated. Philadelphia: P. Blakiston's Son & Company. 1904. (Price, $1.00.)

The author of this manual thinks chemical teaching should be directed to the purpose of aiding pupils in training in the science of prevention, or the diagnosis and treatment of disease, and should consist therefore, in presenting the fundamental principles and their application to the foregoing purpose. This application of the science of chemistry he rightfully groups under the name of *Clinical Chemistry.* This forms the subject matter of a manual, which is the outgrowth of twenty years of teaching in the Long Island College Hospital, and is presented in a most attractive manner in the book now before us.

This second edition having been revised, is made to include such new processes as deserve recognition, some being original. As now arranged and offered the book constitutes one of the best working manuals yet printed on clinical chemistry.

---

Lea's Series of Medical Epitomes. Toxicology. By Edwin Welles Dwight, M.D., Instructor in Legal Medicine, Harvard University. Duodecimo, 298 pages. Series edited by V. C. Pedersen, M.D., Instructor in Surgery at the New York Polyclinic Medical School and Hospital. New York, Philadelphia: Lea Brothers & Company. 1904. (Price, $1.00.)

It is important that every physician who makes any pretense of medical practice, general or special, shall keep himself well prepared to deal with cases of poisoning; also, that every student shall pay diligent heed to the subject that he may be able to cope with emergencies. This epitome will serve to prepare the latter and to refresh the former, keeping both in touch with life-saving and curative methods. It is one of the most important books in this series, and is thoroughly well written.

---

Diseases of the Nose, Throat and Ear and their Accessory Cavities. By Seth Scott Bishop, M.D., Professor of Diseases of the Nose, Throat and Ear in the Illinois Medical College. Third edition, revised and enlarged. Illustrated, including 94 colored lithographs. Octavo, 564 pages. Philadelphia: F. A. Davis Company. 1904. (Price: cloth, $4.00; sheep or half-russia, $5.00 net.)

Several improvements in methods and new apparatus for the treatment of nose and throat affections have prompted a new edition of this well-known treatise and a consequent revision of much of the text. As it now stands it represents the practice of laryngology, rhinology, otology and accessory organs related more or less intimately to these fields, in its most modern fashion. We have on previous occasions, as the several editions have appeared, spoken in favorable terms of Bishop's book, and we now desire to add that it is a condensed, practical treatise which meets the demands of students, practitioners and such physicians as are contemplating the special practice of diseases of the upper air tract.

---

Saunders's Question Compends. Essentials of Bacteriology. By M. V. Ball, M.D., formerly Resident Physician at the German Hospital, Philadelphia. Fifth edition, revised. By Karl M. Vogel, M.D., Assistant Pathologist at the College of Physicians and Surgeons, New York. Duodecimo, 343 pages, illustrated. Philadelphia, New York, London: W. B. Saunders & Company. 1904. ($1.00 net.)

The changes in this topic which have been many and sometimes radical within even a few years, have rendered revision necessary; hence, in this fourth edition of a most useful compend, we find new material relating to advances in tuberculosis, yellow fever, dysentery, bubonic plague, and other infectious diseases, as well as immunity and some other topics. The book well represents the status of bacteriology today, which in the nature of things is changeable and ever changing.

---

Surgery of the Prostrate, Pancreas, Diaphragm, Spleen, Thyroid, and Hydrocephalus. A Historical Review. By Benjamin Merrill Ricketts, M.D., Cincinnati. 1904.

This book is a large octavo printed in double columns, much of it in six-point type and contains 244 pages. It is a reference digest of all available literature, relating to the surgery of the prostate, pancreas, diaphragm, spleen, thyroid, and hydrocephalus, including a recent monograph by the author on the latter subject. It will be observed that it is a work of great value to surgeons, teachers, and writers in particular, though it is useful to any person interested in the literature of these topics. It represents an enormous outlay of time and labor, not to speak of money.

---

Saunders'e Question Compends Essentials of Nervous Diseases and Insanity; Their Symptoms and Treatment. By John C. Shaw, M. D., late Clinical Professor of Diseases of the Mind and Nervous System, Long Island College Hospital Medical School. Fourth edition, revised. By Smith Ely Jelliffe, M.D., Clinical Assistant, Columbia University. Duodecimo, 196 pages, illustrated. Philadelphia, New York, London: W. B. Saunders & Company. 1904. ($1.00 net.)

It is not an easy task to condense the subjects of nervous diseases and insanity into a practical compend. The topics are complex and they are not as susceptible of abridgment or epitomisation as are many medical branches. Nevertheless, we think the authors have produced a cleverly prepared compend that will prove an aid to the student in acquiring a knowledge of the subjects dealt with, provided he uses it in the sense and manner intended—namely, as a stepping-block, so to speak, to a further study of these conditions at the proper time.

---

HANDBOOK OF THE ANATOMY AND DISEASES OF THE EYE AND EAR. By D. B. SAINT JOHN ROOSA, M.D., Professor of Diseases of the Eye and Ear in the New York Post-graduate Medical School, and A. Edward Davis, M.D., Professor of Diseases of the Eye in the New York Post-graduate Medical School. Duodecimo, 300 pages. Philadelphia: F. A. Davis Company. 1904. (Price, $1.00 net.)

A manual of this kind will be welcomed by every physician who is called upon to treat diseases of the eye or ear. Into a compact and limited space the authors have presented the essential principles of treatment of these organs and, besides, sufficient anatomical studies are given to meet the purposes of the book. The volume is intended as a help to either the under- or post-graduate in utilising the material they see in the clinics, that is, to remember it sufficiently well to aid them in their further study and work. A busy doctor who must needs do some work upon the eye and ear, though not a specialist, will find it a reference book of great value. We do not see how a better book for the purposes named could have been made.

---

SAUNDERS'S QUESTION COMPENDS. ESSENTIALS OF ANATOMY. By CHARLES B. NANCREDE, M.D., Professor of Surgery and Clinical Surgery in the University of Michigan, Ann Arbor. Seventh edition, revised. Duodecimo, 419 pages, illustrated. Philadelphia, New York, London: W. B. Saunders & Company. 1904. ($1.00 net.)

An excellent way to begin the study of anatomy is to take this compend into the laboratory and follow its lead in obtaining the first principles. Nancrede is an accomplished anatomist, as well as an experienced teacher. His book has passed to its seventh edition and brought up to the present date in all that relates to anatomical science. Especially has the chapter relating to the nervous system been renewed, while several new ideas relating to other topics have been incorporated, making this edition as complete as it can be.

---

REFRACTION AND HOW TO REFRACT. By JAMES THORINGTON, M.D., Professor of Diseases of the Eye in the Philadelphia Polyclinic. Third edition. Duodecimo, 314 pages, illustrated. Philadelphia: P. Blakiston's Son & Company. 1904. (Price, $1.50.)

The student of refraction,—and we do not mean the undergraduate alone,—will recognise a book that has been a delight to him for six years past. It is now freshened by additions and overhaulings, until it may be said, with a truth, to be the most satisfactory exposition of this special topic that is before the professional public.

Thorington makes his theme interesting even to the general practitioner, not to mention its attractiveness to the ophthalmologist. If Thorington's teachings are once mastered by the novitiate it is quite certain that he will know "how to refract."

---

Saunders's Question Compends. Essentials of Materia Medica and Prescription Writing. By Henry Morris, M.D., College of Physicians, Philadelphia. Sixth edition, revised. By W. A. Bastedo, M.D., Tutor of Materia Medica and Pharmacology at the College of Physicians and Surgeons, New York. Duodecimo, 295 pages. Philadelphia, New York, London: W. B. Saunders & Company. 1905. ($1.00 net.)

The conservative attitude of this compend toward the newer experimental findings, relating to the action of drugs as applied in practice, is wise. In the very nature of things there must be revision and refindings before any new drug can take its appropriate place in the materia medica. Some of the articles have been rewritten and the whole brought forward to the present date. It is an excellent quiz masters' manual as well as students' preparation compend.

---

Transactions of the Ninth Annual Meeting of the American Laryngological, Rhinological and Otological Society, held at Lexington, Ky., April 30, May 1 and 3, 1903. Wendell C. Phillips, M.D., Secretary, New York. Published by the Society. 1903.

The marvelous growth of this society is indicative of the great interest laryngologists manifest in their specialty. Organised in 1896 by scarcely more than a dozen men, it now carries on its rolls a list of members numbering 240, of which number 41 met at Lexington, Ky., and created this excellent volume of transactions. The president's address, by Dr. J. A. Stucky, of Lexington, deals with questions of administration, in addition to which twenty-six papers were read, most of which were discussed with spirit. The book is well made up, but would be improved if it were bound in a substantial manner. Paper covers do not find ready place in the library in these practical days.

---

Thirtieth Annual Report of the Secretary of the State Board of Health of the State of Michigan, for the Fiscal Year Ending June 30, 1902. Henry B. Baker, M.D., Secretary. Lansing: Robert Smith & Company, State Printers and Binders. 1903.

Michigan has been famous for the efficiency of its board of health from the creation of that important body, and especially has the secretary of the board proved an executive officer of great administrative capacity. The annual report is always a marvel of condensed sanitary information, modestly presented, and indicates a thorough watchfulness of the health interests of the state. The dissemination of information through bulletins frequently issued, is an excellent plan which is carried out most admirably by Dr. Baker, and is a most effective way of preventing the spread of contagious and infectious diseases.

